# Effects of Educating Local Government Officers and Healthcare and Welfare Professionals in Suicide Prevention 

**DOI:** 10.3390/ijerph9030712

**Published:** 2012-02-29

**Authors:** Isao Kaniwa, Chiaki Kawanishi, Akira Suda, Yoshio Hirayasu

**Affiliations:** Department of Psychiatry, Yokohama City University School of Medicine, 3-9 Fukuura, Kanazawa-ku, Yokohama 236-0004, Japan; Email: rhythm75kw@yahoo.co.jp (I.K.); sudaakira_ycu@yahoo.co.jp (A.S.); hirayasu@yokohama-cu.ac.jp (Y.H.)

**Keywords:** education, gatekeeper, local government officer, healthcare professional, suicide prevention

## Abstract

Suicide is a major public health issue. In Japan, local governments are responsible for suicide prevention, and local government officers are therefore expected to act as gatekeepers for suicide prevention. In this study, through a questionnaire survey, the authors examined the current knowledge and attitudes concerning suicide prevention among local government officers and healthcare and welfare professionals, and the effects of providing suicide prevention education on their knowledge of and attitudes toward suicide and its prevention. One hundred eighty-three local government officers and 432 healthcare/welfare professionals completed the survey before and after a single education session. Before the session, the local government officers and healthcare/welfare professionals showed mainly positive attitudes toward suicide prevention efforts, with little difference between the two groups. After the training, knowledge and attitudes were further improved for most questionnaire items. Respondents with one or more experiences of suicide prevention training showed significantly more knowledge and positive attitudes before the training than those with no such experience. Moreover, knowledge of depression and having a sympathetic attitude were found to be especially associated with the overall attitude that “suicide can be prevented”. Training in suicide prevention was shown to be effective in promoting appropriate knowledge and attitudes among local government officers and healthcare/welfare professionals who are gatekeepers for preventing suicide. Our findings confirm the importance of suicide prevention education, and will contribute to creating a standard educational program on suicide prevention in Japan.

## 1. Introduction

Suicide is a complicated phenomenon and a major public health issue. While mental disorders clearly contribute to some suicides, other risk factors for suicide include physical health, family history, social isolation, economic circumstances, employment issues, support systems for primary healthcare and welfare, and cultural background [1,2,3,4,5,6,7]. Against this background, individuals who work in the areas of social welfare and healthcare are well placed to act as gatekeepers for suicide prevention, and therefore need appropriate knowledge of suicidal behavior and skills in order to work with suicidal individuals. 

The effects of educating gatekeepers have been reported, but most studies have focused on the education of healthcare professionals [8,9,10]. In Japan, however, local government officers work alongside healthcare and welfare workers as gatekeepers since the Basic Suicide Prevention Law places responsibility for suicide prevention with local governments [11]. Local governments must create policy and implement measures for suicide prevention. In Japanese local governments, officers are placed in charge of counseling for life problems, irrespective of whether they are professionals. Thus, non-professional officers are expected to be gatekeepers who will detect suicidal persons early. Improvement of their skills in relation to suicide prevention is therefore necessary. In this study, we examined the knowledge and attitudes concerning suicide prevention among local government officers and healthcare/welfare professionals, and the effects of providing suicide prevention education on their knowledge of and attitudes toward suicide and its prevention.

## 2. Methods

### 2.1. Participants

Local government officers and healthcare/welfare professionals working for nine local governments and one hospital were targeted in this study. They were asked to participate in this study when a training session on suicide prevention was conducted at their workplace during the period 1 November 2008 to 30 June 2009. A total of 762 individuals participated in this study. 

### 2.2. Procedures

A questionnaire designed by the authors was used to assess knowledge and attitudes concerning suicide. The 10 items consisted of statements about suicide, suicide prevention, and people who attempt suicide to explore the respondents’ knowledge about suicide prevention (four items), and their sympathetic attitude (two items), negative attitude (three items), and overall attitude (one item) toward suicide prevention. Each item was developed considering the nature of suicide and suicidal behaviors as outlined in WHO’s SUPRE publications [12] and was answered using a 5-point scale from 1 (strongly agree) to 5 (strongly disagree). In the current sample, the questionnaire had an internal reliability alpha of 0.63 (10 items). The participants completed the questionnaire immediately prior to the lecture and immediately after it. As training, a single 90-minute lecture on suicide prevention was given by one of the authors (C.K.) at each participating local government and hospital site. The contents of the lecture are shown in Appendix I.

### 2.3. Ethics

The study protocol was approved by the Ethics Committee of Yokohama City University School of Medicine.

### 2.4. Statistical Analysis

Statistical analysis was conducted using PASW for Windows version 18.0 (SPSS Inc, Chicago, IL, USA). The chi-square test was used to explore differences in knowledge and attitudes in relation to sex, occupation, and previous education, and the Kruskal-Wallis test was used to determine the differences in relation to age. The paired t-test was used to compare differences in knowledge and attitudes between before and after the lecture. Further, categorical regression analysis was performed to determine differences in overall attitude before and after the lecture. In the categorical regression model, we used Q10 (“Suicide can be prevented”) as the dependent variable, and the other 9 items as independent variables. A probability level of *p* < 0.05 was considered statically significant.

## 3. Results

### 3.1. Respondent Demographics

From the total 762 participants who attended the training, 646 (84.8%) answered the questionnaire before the training started. The demographics of the respondents are shown in Table 1. The respondents were 221 (34.2%) males and 425 (65.8%) females, with a mean age of 42.4 (SD, 12.3) years and range of 20 to 76 years. In regard to occupation, 432 (66.9%) were healthcare/welfare professionals, 183 (28.3%) were local government officers, and 31 (4.8%) were not determined. One hundred fifty-five (24.2%) had one or more experiences of suicide prevention education, 300 (46.4%) had never attended an education session before, and the remaining 190 (29.4%) did not respond to the question.

**Table 1:** Respondent demographics.

		Total (N = 646)	Male (N = 221)	Female (N = 425)
		n (%)	n (%)	n (%)
Age	20–29	130 (20.1)	29 (13.1)	101 (23.8)
	30–39	136 (21.1)	43 (19.5)	93 (21.9)
	40–49	148 (22.9)	50 (22.6)	98 (23.1)
	50–59	183 (28.3)	86 (38.9)	97 (22.8)
	60–69	30 (4.6)	9 (4.1)	21 (4.9)
	≥70	4 (0.6)	2 (0.9)	2 (0.5)
	N.D.	15 (2.3)	2 (0.9)	13 (3.1)
Occupation	HCP	432 (66.9)	96 (43.4)	336 (79.1)
	LGO	183 (28.3)	119 (53.8)	64 (15.1)
	N.D.	31 (4.8)	6 (2.7)	25 (5.9)
Previous education	Ed	156 (24.1)	49 (22.2)	107 (25.2)
	Non-Ed	300 (46.4)	76 (34.4)	224 (52.7)
	N.D.	190 (29.4)	96 (43.4)	94 (22.1)

HCP, healthcare/welfare professional; LGO, local government officer; Ed, respondents with one or more experience of education in suicide prevention; Non-Ed, respondents with no experience of education in suicide prevention; N.D., not determined.

### 3.2. Knowledge and Attitudes by Demographics Before the Training

Table 2 shows the proportion of answers to each of the questionnaire statements by respondent demographics. Overall, most respondents showed that they felt efforts at preventing suicide could be successful and that they had appropriate knowledge of suicide (Q1, Q3, Q5, Q6, and Q10). However, the majority of respondents did have some inappropriate knowledge and attitudes regarding suicide, as shown in their answers to 3 items (Q4, Q8, and Q9). In addition, many respondents were unsure how to answer 2 items (Q2 and Q7).

**Table 2:** Knowledge and attitude toward suicide prevention by demographics before the training.

	N	Stronglyagree	Agree	Not sure	Disagree	Stronglydisagree	χ²	*P*-value
		%	%	%	%	%		
**(Knowledge)**								
Q1 Suicide can be prevented by resolving life stressors.								
Total	645	13.0	52.7	25.4	7.6	1.2		
Male/Female	221/424	14.0/12.5	53.4/52.4	22.6/26.9	8.1/7.3	1.8/0.9	2.340	0.673
HCP/LGO	431/183	11.4/16.9	51.3/56.3	26.9/21.3	9.0/4.4	1.4/1.1	9.027	0.060
Ed/Non-Ed	155/300	14.2/13.3	57.4/48.7	18.1/29.3	9.0/7.3	1.3/1.3	7.036	0.134
Q2 The majority of people who commit suicide have mental illness.								
Total	646	9.1	28.0	37.6	20.6	4.6		
Male/Female	221/425	10.0/8.7	31.2/26.4	34.8/39.1	18.6/21.6	5.4/4.2	3.289	0.511
HCP/LGO	432/183	9.3/9.3	27.8/28.4	38.0/37.7	21.5/18.6	3.5/6.0	2.522	0.641
Ed/Non-Ed	156/300	12.2/6.7	40.4/19.7	32.1/40.7	12.2/28.0	3.2/5.0	34.258	<0.001
Q3 Having appropriate knowledge about depression is crucial to suicide prevention efforts.								
Total	646	14.7	54.0	23.2	6.8	1.2		
Male/Female	221/425	11.8/16.2	58.4/51.8	20.4/24.7	7.7/6.4	1.8/0.9	5.601	0.231
HCP/LGO	432/183	16.0/9.8	52.3/59.6	23.1/23.5	6.9/6.6	1.6/0.5	5.836	0.212
Ed/Non-Ed	156/300	23.7/12.7	52.6/49.7	19.2/26.3	2.6/10.0	1.9/1.3	17.800	0.001
Q4 A person who commits suicide does so calmly, in a rational manner.								
Total	645	0.9	7.8	32.2	43.7	15.3		
Male/Female	220/425	1.4/0.7	10.0/6.6	30.9/32.9	40.9/45.2	16.8/14.6	4.110	0.391
HCP/LGO	431/183	0.5/2.2	7.7/8.2	32.5/32.8	45.0/41.0	14.4/15.8	4.609	0.330
Ed/Non-Ed	155/300	0.6/1.0	7.1/9.0	31.0/32.0	42.6/43.0	18.7/15.0	1.493	0.828
**(Sympathetic attitude)**								
Q5 A person who attempts suicide is essentially crying out for help.								
Total	644	45.7	38.5	13.5	1.9	0.5		
Male/Female	219/425	45.7/45.6	37.0/39.3	14.2/13.2	2.7/1.4	0.5/0.5	1.671	0.796
HCP/LGO	431/182	45.5/45.1	39.9/36.8	13.0/14.3	1.2/3.3	0.5/0.5	3.754	0.440
Ed/Non-Ed	156/299	59.0/38.8	29.5/41.1	10.3/17.4	0.6/2.0	0.6/0.7	17.612	0.001
Q6 Active listening may help a person who is at risk of attempting suicide.								
Total	626	53.4	40.4	5.6	0.6	0.0		
Male/Female	212/414	48.1/56.0	44.3/38.4	7.1/4.8	0.5/0.7	0.0/0.0	4.276	0.233
HCP/LGO	417/180	54.7/51.1	39.8/41.7	4.8/6.7	0.7/0.6	0.0/0.0	1.277	0.735
Ed/Non-Ed	152/288	55.3/48.3	41.4/43.4	3.3/7.3	0.0/1.0	0.0/0.0	5.331	0.149
**(Negative attitude)**								
Q7 Suicide is an irresponsible act that cuts short life.								
Total	644	7.6	17.7	41.0	23.0	10.7		
Male/Female	221/423	10.0/6.4	19.0/17.0	31.7/45.9	26.2/21.3	13.1/9.5	13.265	0.010
HCP/LGO	430 /183	6.5/10.4	20.0 /14.2	41.4/38.3	21.6/25.1	10.5/12.0	6.161	0.187
Ed/Non-Ed	155/299	7.1/8.7	8.4/22.7	37.4/41.8	30.3/19.1	16.8 /7.7	26.049	<0.001
Q8 People confiding that they are suicidal should be remonstrated with and encouraged to think differently.								
Total	644	2.0	4.7	23.4	39.9	30.0		
Male/Female	219/425	4.1/0.9	6.8/3.5	28.3/20.9	33.8/43.1	26.9/31.5	18.081	0.001
HCP/LGO	431/182	0.7/4.9	2.8/9.9	18.3/34.6	44.1/31.3	34.1/19.2	54.368	<0.001
Ed/Non-Ed	156/299	0.6/1.7	3.2/5.0	16.7/24.7	41.7/41.1	37.8/27.4	8.221	0.084
Q9 Once a person has made up his/her mind about committing suicide, no-one can stop it.								
Total	625	1.1	5.4	25.4	45.4	22.6		
Male/Female	211/414	1.4/1.0	5.7/5.3	24.2/26.1	44.1/46.1	24.6 /21.5	1.241	0.871
HCP/LGO	417/179	1.2/1.1	6.5/3.9	24.5/27.4	46.3/44.1	21.6/23.5	2.197	0.700
Ed/Non-Ed	152/288	1.3/1.0	4.6/6.9	23.0/28.5	44.7/42.4	26.3/21.2	3.336	0.503
**(Overall attitude)**								
Q10 Suicide can be prevented.								
Total	625	33.4	52.5	13.1	1.0	0.0		
Male/Female	212/413	34.4/32.9	50.9/53.3	13.7/12.8	0.9/1.0	0.0/0.0	0.317	0.957
HCP/LGO	416/180	32.0 /35.6	54.8/47.8	12.3/16.1	1.0/0.6	0.0/0.0	3.302	0.347
Ed/Non-Ed	152/288	44.7/27.1	48.7/53.1	6.6/18.1	0.0/1.7	0.0/0.0	21.663	<0.001

Chi-square test for categorical values; HCP, healthcare/welfare professional; LGO, local government officer; Ed, respondents with one or more experience of education in suicide prevention; Non-Ed, respondents with no experience of education in suicide prevention.

The significant differences seen were as follows. Significantly more female than male respondents answered “not sure” to Q7 (“Suicide is an irresponsible act that cuts short life”; χ² = 13.265, *p* = 0.010). Also, significantly more female than male respondents disagreed with the statement in Q8 (“People confiding that they are suicidal should be remonstrated with and encouraged to think differently”; χ² = 18.081, *p* = 0.001), and significantly more healthcare/welfare professionals than local government officers disagreed with this statement (χ² = 54.368, *p* < 0.001). 

Moreover, significantly more respondents with experience of education in suicide prevention compared to those without such experience agreed with the statements in Q2 (“The majority of people who commit suicide have mental illness”; χ² = 34.258, *p* < 0.001), Q3 (“Having appropriate knowledge about depression is crucial to suicide prevention efforts”; χ² = 17.800, *p* = 0.001), Q5 (“A person who attempts suicide is essentially crying out for help”; χ² = 17.612, *p* = 0.001), and Q10 (“Suicide can be prevented”; χ² = 21.663, *p* < 0.001). On the other hand, there were significantly more respondents with experience of education who disagreed with the statement in Q7 (“Suicide is an irresponsible act that cuts short life”; χ² = 26.049, *p* < 0.001) compared to those without such experience.

### 3.3. Changes in Knowledge and Attitudes after the Training

Among the 646 respondents, 342 (52.9%) completed the questionnaire after the training session. Changes in their knowledge and attitudes concerning suicide are summarized in Table 3. 

**Table 3:** Change in knowledge and attitude toward suicide prevention following the training.

	Pre-test		Post-test		Paired differences			T-value	DF	*P*-value
	Mean	SD	Mean	SD	Mean	SD	SEM			
**(Knowledge)**										
Q1	2.26	0.85	2.26	0.96	0.00	0.99	0.05	0.000	341	1.000
Q2	2.73	1.02	1.56	0.75	1.17	1.15	0.06	18.834	341	<0.001
Q3	2.20	0.78	1.72	0.66	0.47	0.80	0.04	10.975	341	<0.001
Q4	3.69	0.84	4.03	1.01	−0.34	0.99	0.05	−6.423	340	<0.001
**(Sympathetic attitude)**										
Q5	1.67	0.77	1.40	0.68	0.27	0.73	0.04	6.970	338	<0.001
Q6	1.54	0.60	1.34	0.55	0.20	0.60	0.03	6.039	336	<0.001
**(Negative attitude)**										
Q7	3.25	1.03	3.73	1.04	−0.48	0.99	0.05	−8.948	339	<0.001
Q8	3.89	0.99	4.31	0.92	−0.42	0.91	0.05	−8.590	336	<0.001
Q9	3.92	0.80	4.17	0.84	−0.25	0.83	0.05	−5.519	335	<0.001
**(Overall attitude)**										
Q10	1.77	0.65	1.50	0.57	0.27	0.59	0.03	8.345	336	<0.001

Paired t-test compared with pre-test.

Significant improvement was seen for three of the four items concerning appropriate knowledge, namely, Q2 (t (341) = 18.834, *p* < 0.001), Q3 (t (341) = 10.975, *p* < 0.001), and Q4 (t (340) = 6.423, *p* < 0.001), but not for Q1. Significant improvement was also seen in regard to sympathetic attitude, for Q5 (t (338) = 6.970, *p* < 0.001), and Q6 (t (336) = 6.039, *p* < 0.001). As for negative attitude, significant improvement was seen for all three items, namely, Q7 (t (339) = 8.948, *p* < 0.001), Q8 (t (336) = 8.590, *p* < 0.001), and Q9 (t (335) = 5.519, *p* < 0.001). Lastly, as to overall attitude determined by Q10, attitude was significantly improved (t (336) = 8.345, *p* < 0.001).

### 3.4. Factors Influencing Overall Attitude

Table 4 shows the results of categorical regression analysis performed to determine factors influencing the overall attitude that “suicide prevention is possible”. Six variables (Q2, Q3, Q5, Q6, Q7, and Q9) were found to be significantly associated with this overall attitude (Q10). Among them, relatively influential variables were Q3 (β = 0.246), Q5 (β = 0.181), Q6 (β = 0.183), and Q9 (β = −0.190).

**Table 4:** Factors influencing respondents’ overall attitude.

	β	SE	DF	F	*P*-value
**(Knowledge)**					
Q1	0.072	0.054	1	1.806	0.180
Q2	0.109	0.042	2	6.781	0.001
Q3	0.246	0.046	2	28.164	<0.001
Q4	−0.058	0.085	1	0.460	0.498
**(Sympathetic attitude)**					
Q5	0.181	0.078	3	5.384	0.001
Q6	0.183	0.068	2	7.291	0.001
**(Negative attitude)**					
Q7	0.103	0.035	3	8.712	<0.001
Q8	−0.025	0.059	1	0.187	0.665
Q9	−0.190	0.066	3	8.368	<0.001

Categorical regression analysis used Q10 (Overall attitude) as the dependent variable and the other nine items as independent variables.

## 4. Discussion

The positive effects of educational programs on suicide prevention for healthcare professionals have been reported. In the Gotland study, educating general practitioners about depression resulted in a decreased suicide rate among their patients [8,13]. In the STORM project, a brief educational program aimed at improving assessment and management of known suicide risks raised the confidence of front-line health professionals in contact with suicidal individuals [9,14]. However, few studies have explored the effects of such education for local government officers. Because local government officers in Japan carry out reception work and consultation services for citizens, and are mandated by law to do so, they play a gatekeeper’s role in preventing suicide. 

Looking at our results, both the local government officers and healthcare/welfare professionals answered most questionnaire items appropriately before the training, which is encouraging. Knowledge and attitudes were significantly improved immediately after the training session for all items except Q1, concerning preventing suicide by resolving life stressors. No significant improvement was observed for this item because, before the training, many respondents already held the statement that “suicide can be prevented by solving life stressors” to be true, as shown by their pre-training answers to Q1.

After the training, there was no significant difference seen between the local government officers and healthcare/welfare professionals only for Q8 (“People confiding that they are suicidal should be remonstrated with and encouraged to think differently”). More healthcare/welfare professionals responded appropriately to this statement than local government officers, who tended to respond with uncertainty or incorrectly to it. This is likely because healthcare/welfare professionals might have had many opportunities to talk with suicidal individuals and be better trained in suicide prevention.

When looking at previous experience of education in suicide prevention, respondents with one or more experiences of such training more frequently answered five of the questionnaire items appropriately. Alongside “the short-term educational effect” shown by participants following the training session given in this study, it is interesting to note this “long-term educational effect” as well. The central dogma that “suicide can be prevented” was found to be influenced by multiple factors, but particularly “knowledge of depression” and “sympathetic attitudes”. These items are therefore topics that need to be covered in suicide prevention education. 

There are some limitations in our study. First, the sample size of the local government officers was relatively small. Second, the response rate after the training session was low and might have affected the results. Third, because the content of previous education on suicide prevention provided to some respondents is unknown, long-term educational effect on suicide prevention could not be assessed. Lastly, the self-reported questionnaire used might not reflect the respondents’ actual knowledge and attitudes.

## 5. Conclusions

In conclusion, this study revealed the basic knowledge and attitudes of both local government officers and healthcare/welfare professionals acting as gatekeepers in Japan, showed a positive educational effect of specific training on suicide prevention, and revealed the factors that need to be covered by educational programs. Our findings can contribute to creating a standard educational program for suicide prevention in Japan. Our results also show that repeated opportunities for education are important as those with prior experience of specific training showed improved knowledge after further training. Increasing knowledge changes attitudes, and increasing communication and intervention skills decreases people’s feelings of difficulty or anxiety about providing support for suicide prevention. The authors believe that participation in workshop-style education which present case studies and includes skill improvement training is necessary for local government officers in Japan who lack experience of communicating directly with suicidal individuals.

## Appendix

Lecture session (90 min)

(1) Epidemiology of suicide

(2) Risk factors and process of suicide

(3) Depression and other mental illnesses

(4) Psychology of suicidal individuals

(5) Suicide prevention strategy and examples

(6) Communicating with suicidal individuals
